# Human immune globulin treatment controls Zika viremia in pregnant rhesus macaques

**DOI:** 10.1371/journal.pone.0266664

**Published:** 2022-07-14

**Authors:** Dawn M. Dudley, Michelle R. Koenig, Laurel M. Stewart, Matthew R. Semler, Christina M. Newman, Phoenix M. Shepherd, Keisuke Yamamoto, Meghan E. Breitbach, Michele Schotzko, Sarah Kohn, Kathleen M. Antony, Hongyu Qiu, Priyadarshini Tunga, Deborah M. Anderson, Wendi Guo, Maria Dennis, Tulika Singh, Sierra Rybarczyk, Andrea M. Weiler, Elaina Razo, Ann Mitzey, Xiankun Zeng, Jens C. Eickhoff, Emma L. Mohr, Heather A. Simmons, Michael K. Fritsch, Andres Mejia, Matthew T. Aliota, Thomas C. Friedrich, Thaddeus G. Golos, Shantha Kodihalli, Sallie R. Permar, David H. O’Connor

**Affiliations:** 1 Department of Pathology and Laboratory Medicine, University of Wisconsin-Madison, Madison, WI, United States of America; 2 Department of Comparative Biosciences, University of Wisconsin-Madison, Madison, WI, United States of America; 3 Wisconsin National Primate Research Center, University of Wisconsin-Madison, Madison, WI, United States of America; 4 Department of Radiology, University of Wisconsin-Madison, Madison, WI, United States of America; 5 Department of Obstetrics and Gynecology, University of Wisconsin-Madison, Madison, WI, United States of America; 6 Emergent BioSolutions, Canada Inc., Winnipeg, MB, Canada; 7 Department of Pharmacology and Cancer Biology, Duke University Medical Center, Durham, NC, United States of America; 8 Department of Pediatrics and Human Vaccine Institute, Duke University Medical Center, Durham, NC, United States of America; 9 Department of Pediatrics, University of Wisconsin-Madison, Madison, WI, United States of America; 10 United States Army Medical Research Institute of Infectious Diseases, Fort Detrick, Frederick, MD, United States of America; 11 Biostatistics and Medical Informatics, University of Wisconsin-Madison, Madison, WI, United States of America; 12 Department of Veterinary and Biomedical Sciences, University of Minnesota, Twin Cities, St. Paul, MN, United States of America; 13 Department of Pathobiological Sciences, University of Wisconsin-Madison, Madison, WI, United States of America; 14 Department of Pediatrics, Weill Cornell Medicine, New York, NY, United States of America; University of Pittsburgh, UNITED STATES

## Abstract

There are currently no approved drugs to treat Zika virus (ZIKV) infection during pregnancy. Hyperimmune globulin products such as VARIZIG and WinRho are FDA-approved to treat conditions during pregnancy such as Varicella Zoster virus infection and Rh-incompatibility. We administered ZIKV-specific human immune globulin as a treatment in pregnant rhesus macaques one day after subcutaneous ZIKV infection. All animals controlled ZIKV viremia following the treatment and generated robust levels of anti-Zika virus antibodies in their blood. No adverse fetal or infant outcomes were identified in the treated animals, yet the placebo control treated animals also did not have signs related to congenital Zika syndrome (CZS). Human immune globulin may be a viable prophylaxis and treatment option for ZIKV infection during pregnancy, however, more studies are required to fully assess the impact of this treatment to prevent CZS.

## Introduction

In utero Zika virus (ZIKV) infection causes devastating congenital outcomes in 5–10% of fetuses [1, 2]. Even though ZIKV has receded from public attention, the risk of future outbreaks looms large. The *Aedes aegypti* mosquito vector that primarily transmits ZIKV is found in 61 countries where the virus has never been documented [3]. Even in countries with documented ZIKV transmission, seroprevalence is often low (~10%) [4, 5]. Together, these data suggest that there are many ZIKV-naive individuals at risk for future infections. In addition, durability of ZIKV immunity is unknown for individuals living in regions that have already experienced outbreaks. If ZIKV outbreaks mimic those of the genetically related dengue virus (DENV), endemo-epidemic outbreaks are likely, motivating the development of medical countermeasures that could be deployed quickly in future outbreaks [6]. To prevent congenital Zika syndrome (CZS), which includes conditions like microcephaly, congenital joint contractures, ocular abnormalities, and auditory deficits, it is important to establish treatment and prevention approaches now.

There are no FDA-approved therapies to treat or prevent ZIKV infection. Promising vaccines have made it through phase I and into phase II clinical trials, but are stalled due to lack of ongoing ZIKV transmission [7, 8]. Vaccination before conception in a pregnant rhesus macaque model reduced the risk of CZS and appeared to prevent detectable virus in the fetus, despite the fact that it did not prevent maternal viremia in over half of the animals [9]. However, many ZIKV vaccine candidates use a live attenuated virus backbone, which is often contraindicated for use during pregnancy due to a theoretical risk to the fetus [10, 11]. Safe biological products that can be used early during pregnancy to prevent or treat ZIKV and mitigate the impact of congenital effects would be especially desirable.

Hyperimmune globulin (HIG) is one such biological product. HIG is produced by isolating mature, high-affinity IgG antibodies from individuals naturally exposed to a pathogen. Hyperimmune globulin is FDA-approved to treat Varicella Zoster virus infection (VARIZIG), Rh-incompatibility (Rhophylac, WinRho), and other conditions such as immune thrombocytopenia (WinRho SDF) in pregnant women [12, 13]. Hyperimmune globulin has also been tested to treat other emerging infectious diseases for which treatment options are limited such as H1N1 influenza, Ebola, and SARS-CoV-2 [14–16]. Lastly, HIG contains both neutralizing and binding antibodies that may mediate alternative virus-specific mechanisms to control viral replication, such as antibody-dependent cellular cytotoxicity (ADCC).

Fortuitously, antibodies (Abs), especially neutralizing antibodies (NAbs), are considered the primary correlate of immune protection for ZIKV based on protective vaccines tested in rhesus macaques [17, 18]. Immunoglobulins isolated from vaccinated animals were sufficient to protect naive animals from ZIKV infection [17]. We have shown that PRNT50 values of >2.75 Log10 serum dilution, in animals exposed to ZIKV approximately 2 years before rechallenge, were sufficient for protection from re-infection [19]. Similarly, Abbink, et al. showed that a 50% microneutralization log titer of 2.0–2.1 following vaccination was the required threshold for protection in their study [18]. Lastly, monoclonal antibodies (mAbs) have been tested as passive immunization in nonhuman primates. One study showed that mAbs were effective at blunting viral replication, but ZIKV can rapidly develop escape mutations in the presence of a single Ab thereby necessitating treatment with at least two different mAbs [20]. Even with two mAbs, viremia was not prevented in this study. In another study testing a cocktail of 3 mAbs 24 hours before infection, 4 of 4 animals were protected from detectable virus in the blood. In pregnant rhesus macaques, this cocktail of mAbs was provided at 3 days post-infection and cleared virus from the serum in 2 of 3 animals, but virus was still detectable in the amniotic fluid of two of the animals, though not in the fetus at the time of birth a few weeks later [21].

In this study, we treat eight pregnant Indian-origin rhesus macaques with either human-derived ZIKV-specific IgG (ZIKV-IG) or human-derived placebo-IG one day after ZIKV infection around gestational day (GD) 45 (end of first trimester). In macaques each trimester is ~55 days and full term is considered 165 days. We monitor plasma viral load in the dams, fetal growth, pharmacokinetics of the infused ZIKV-IG, vertical transmission, birth defects, and histopathology in the infant at the time of birth by cesarean section at ~GD 155. ZIKV-IG treatment dramatically reduces plasma ZIKV viremia in the dams after infusion while placebo-IG does not. No ZIKV-associated birth defects nor ZIKV RNA are found in the infants at the time of birth in either the ZIKV-IG group or the placebo-IG group.

## Results

### Experimental design

**Fig 1:**
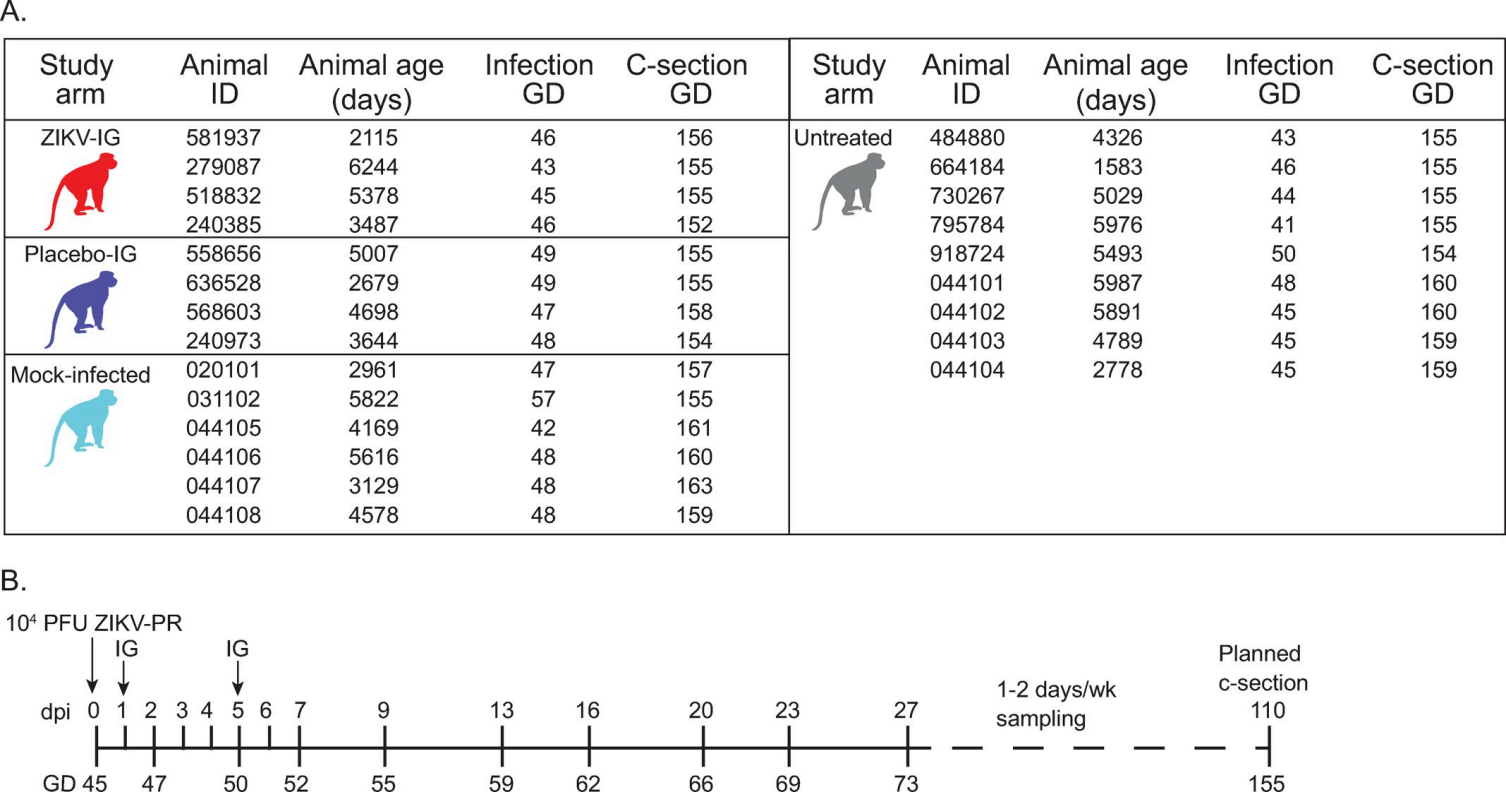
Animal demographics and study timeline. (**A**) Animal ID, animal age at the time of ZIKV or mock inoculation, gestational day (GD) at the time of infection, and gestational day at the time of cesarean section (C-section). Each study arm is represented in subsequent graphs by the color shown in the monkey for each group. (**B**) Timeline representing the day of inoculation, treatment, blood sampling (ticks on the line), and C-section for animals in this study relative to either days post-inoculation (dpi) or gestational day (GD). Blood sampling occurred as shown through 27 dpi and then was twice weekly until two consecutive time points were negative for ZIKV RNA and then sampling was once weekly.

Eight pregnant rhesus macaques were infected subcutaneously with 1 x 10^4^ plaque forming units (PFU) of ZIKV (PRVABC59, see methods) at gestational day 45 (+/- 4 days) (Fig 1A). Four animals were treated at 1 and 5 days post-infection (dpi) with 50mg/kg of human immune globulin (HIG) isolated from ZIKV-seropositive plasma donors (ZIKV-IG). The other four animals were treated with 50mg/kg of HIG from ZIKV-naive individuals (placebo-IG) (Fig 1B). Historical, mock-infected (n = 6) and untreated (n = 9) pregnant macaques infected with the same strain, dose, and route of ZIKV were used as additional control groups for various analyses [22, 23] (Fig 1A). Pregnant animals receiving ZIKV-IG or placebo-IG were monitored throughout pregnancy and a cesarean section was performed at GD155 (+/- 4 days) (Fig 1B). Infants were euthanized and an extensive post-mortem examination with sample collection was performed.

### ZIKV-IG clears maternal plasma ZIKV viremia at 1 day post infusion

**Fig 2:**
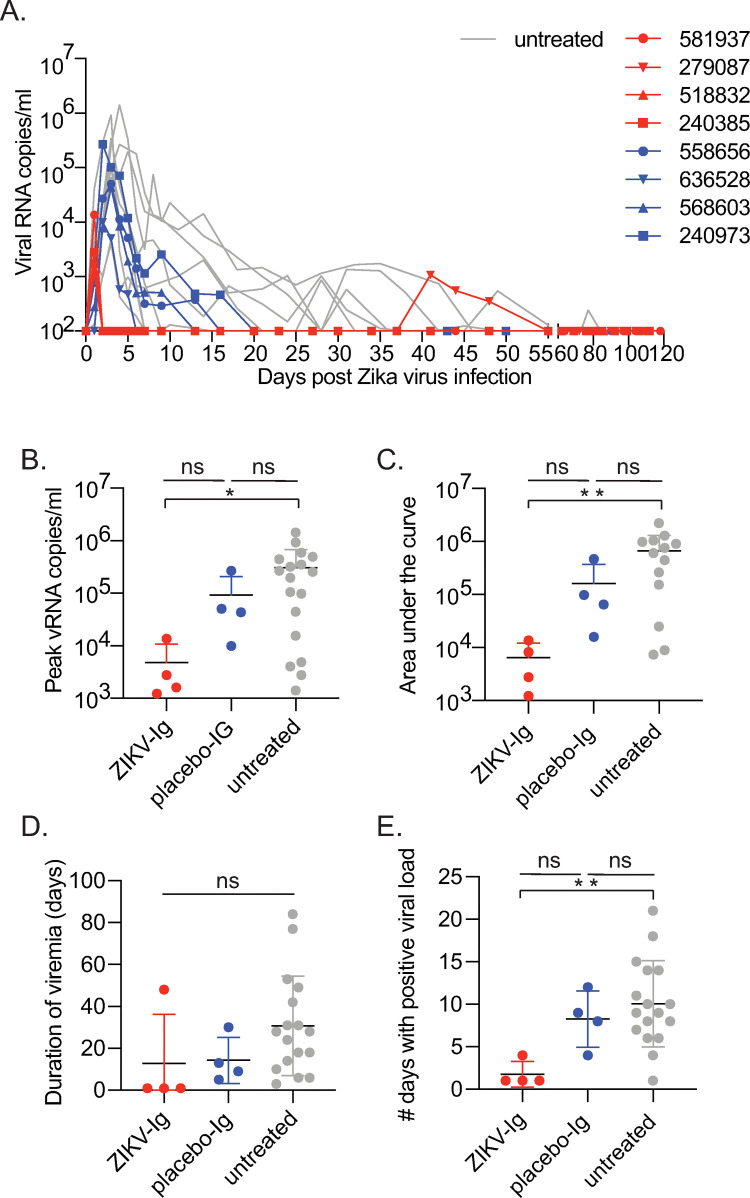
Dam viral load analysis between ZIKV-IG, placebo-IG, and untreated animals. (**A**) Longitudinal plasma viral load for animals across each group. Red = ZIKV-IG-treated, blue = placebo-IG-treated, gray = untreated. The x-axis is set to the limit of quanti of the assay (100 copies/ml). (**B**) Peak viral load was statistically different for each group shown with the mean and standard deviation (Kruskall-Wallis chi-squared = 7.1263, df = 2, p = 0.03). ZIKV-IG-treated dams had lower viremia levels than untreated controls (p = 0.02; *p<0.05), but placebo-IG-treated animals did not (p = 0.89) and ZIKV-IG- and placebo-IG were also not statistically different (p = 0.130). (**C**) Area under the curve was statistically different between the ZIKV-IG group and untreated animals (p = 0.01; **p<0.01) but the placebo-IG group was not (p = 0.60). ZIKV-IG- and placebo-IG-treated groups were not statistically different from each other (p = 0.1859). The graph shows the mean and standard deviation. (**D**) Duration of viremia, as shown with the mean and standard deviation, was not different between groups (Kruskal-Wallis chi-squared = 5.05, df = 2, p = 0.08). (**E**) The total number of days with a positive viral load was different between the ZIKV-IG and untreated groups (p = 0.01) but not between placebo-IG and untreated animals (p = 1.00). ZIKV-IG- and placebo-IG-treated groups were not statistically different from each other (p = 0.085). Data shown as the mean with standard deviation.

Following ZIKV infection and HIG infusion, animals were carefully monitored for clinical signs of infection and reaction to HIG infusion. Animals did not experience weight loss, fever, or other signs of infection and no reaction to HIG infusion (S1 File and S1 Fig). ZIKV viral loads in plasma were measured by qRT-PCR daily for the first week after inoculation and then once or twice weekly until the end of pregnancy. All ZIKV-IG-treated animals had detectable virus at 1 dpi (1,225–3,620 copies vRNA/ml) prior to HIG administration and all placebo-IG-treated animals had detectable viral RNA by 1 or 2 dpi (Fig 2A). By 2 dpi, all ZIKV-IG-treated animals had levels of virus below the limit of quantification of the viral load assay (100 copies/ml) (Fig 2A). All placebo-IG-treated animals continued to have detectable viremia for 5–16 dpi.

One ZIKV-IG-treated animal (279087) exhibited viral recrudescence, with plasma viral loads detectable at 1x10^2^-1x10^3^ copies/ml from 41–48 dpi. This animal initially tested negative for 15 time points starting at 2 dpi and then controlled viremia after the recrudescence to below detection (<100 copies/ml) for the rest of the pregnancy (Fig 2A). Peak viral load differed significantly between groups (Fig 2B). Specifically, ZIKV-IG-treated dams had lower viremia levels compared to untreated controls (p = 0.02), but placebo-IG-treated animals were not significantly different from ZIKV-IG-treated (p = 0.130) or untreated controls (p = 0.89). Lack of statistical significance between ZIKV-IG and placebo-IG animals is due to small group sizes. When comparing each group to the larger untreated control group, ZIKV-IG-treated animals are statistically different while placebo-IG-treated animals are not. Area under the curve (AUC) was lower in ZIKV-IG-treated compared to untreated animals, but not between placebo-IG- and ZIKV-IG-treated or untreated animals (Fig 2C). Duration of plasma viremia, defined as the last time point of detectable viremia, was not significantly different between the ZIKV-IG, placebo-IG and untreated groups (Fig 2D). This lack of significance was due to the single animal exhibiting viral recrudescence, pushing the mean duration of viremia for the group to something similar to the placebo-IG group, even though most animals had one day of viremia. The total number of timepoints with detectable vRNA was significantly different between the ZIKV-IG-treated and untreated groups (p = 0.01), but was the same between placebo-IG and untreated animals (p = 1.000) and not significantly different between ZIKV-IG- and placebo-IG-treated animals (p = 0.085) (Fig 2E). Altogether, ZIKV-IG significantly decreased peak viral load and the number of ZIKV vRNA positive days in the treated dams compared to untreated animals.

### Antibody dynamics and pharmacokinetics of HIG in pregnant macaques

**Fig 3:**
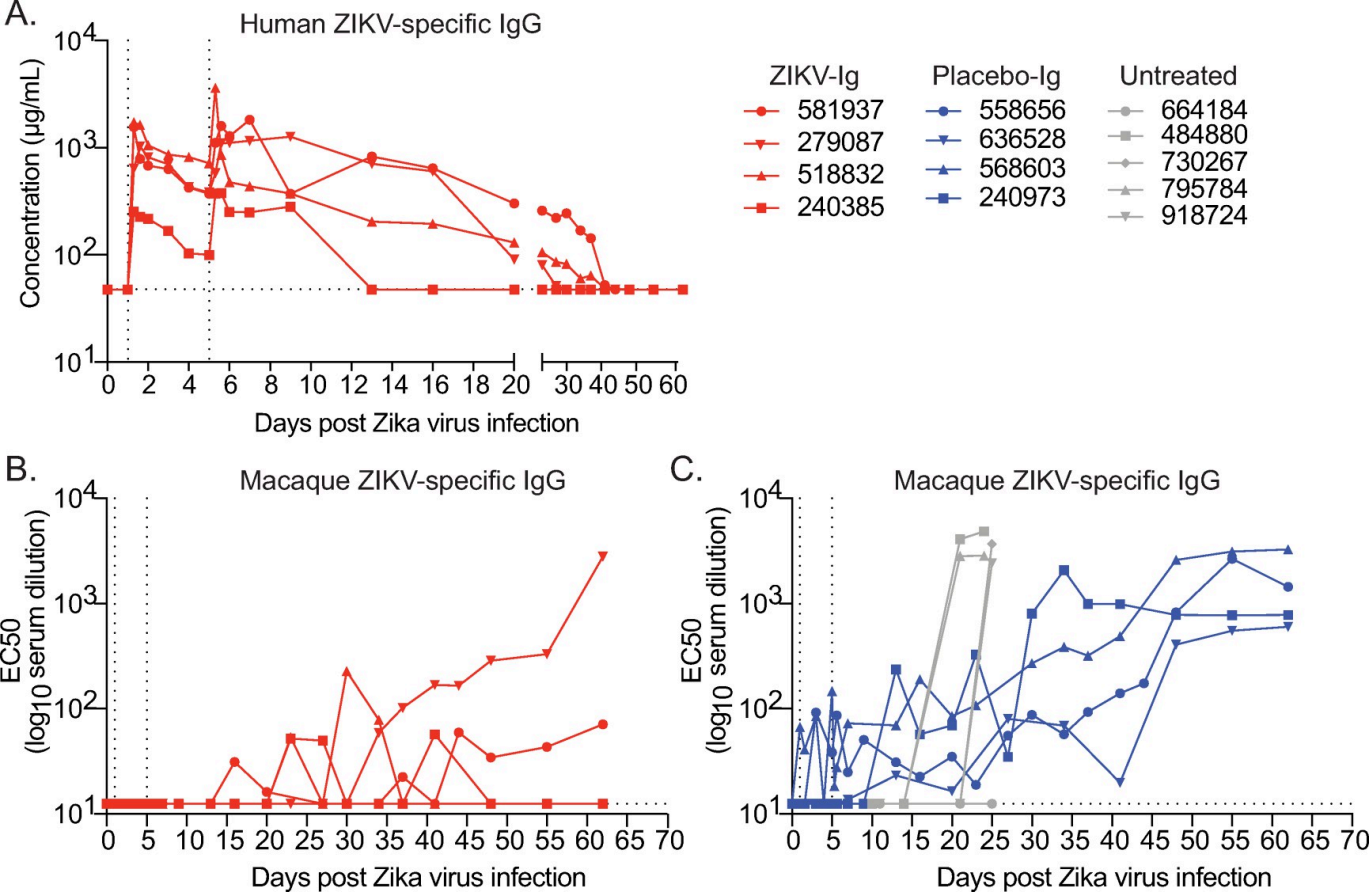
Measurement of human or macaque-specific binding antibody titers by whole virion binding ELISA. (A) Concentration of human ZIKV-specific IgG antibodies measured longitudinally in ZIKV-IG-treated animals. (B) EC50 of macaque ZIKV-specific IgG antibodies in ZIKV-IG-treated animals. (C) EC50 of macaque ZIKV-specific IgG antibodies in placebo-IG and untreated animals. Dotted lines represent the limit of detection for each assay. Vertical dotted lines indicate the time points of IgG infusion.

ZIKV-specific binding antibody (bAb) titers were followed longitudinally throughout pregnancy to monitor the pharmacokinetics (PK) of the antibody infusions and track de novo antibody responses. ZIKV-specific bAb titers were measured by whole virion binding ELISA using captured PRVABC59 virions. Human and macaque-specific anti-ZIKV IgG were distinguished by species-specific secondary antibodies. As expected, peak human ZIKV-specific IgG antibody levels occurred 1 hour after each infusion, which occurred at 1 and 5 dpi (Fig 3A). Only two ZIKV-IG-treated animals developed durable macaque ZIKV-specific Ab responses still detectable at the end of the study (Fig 3B) while all four placebo-IG-treated animals developed a durable Ab response (Fig 3C). Detailed results are presented in S1 File.

**Fig 4:**
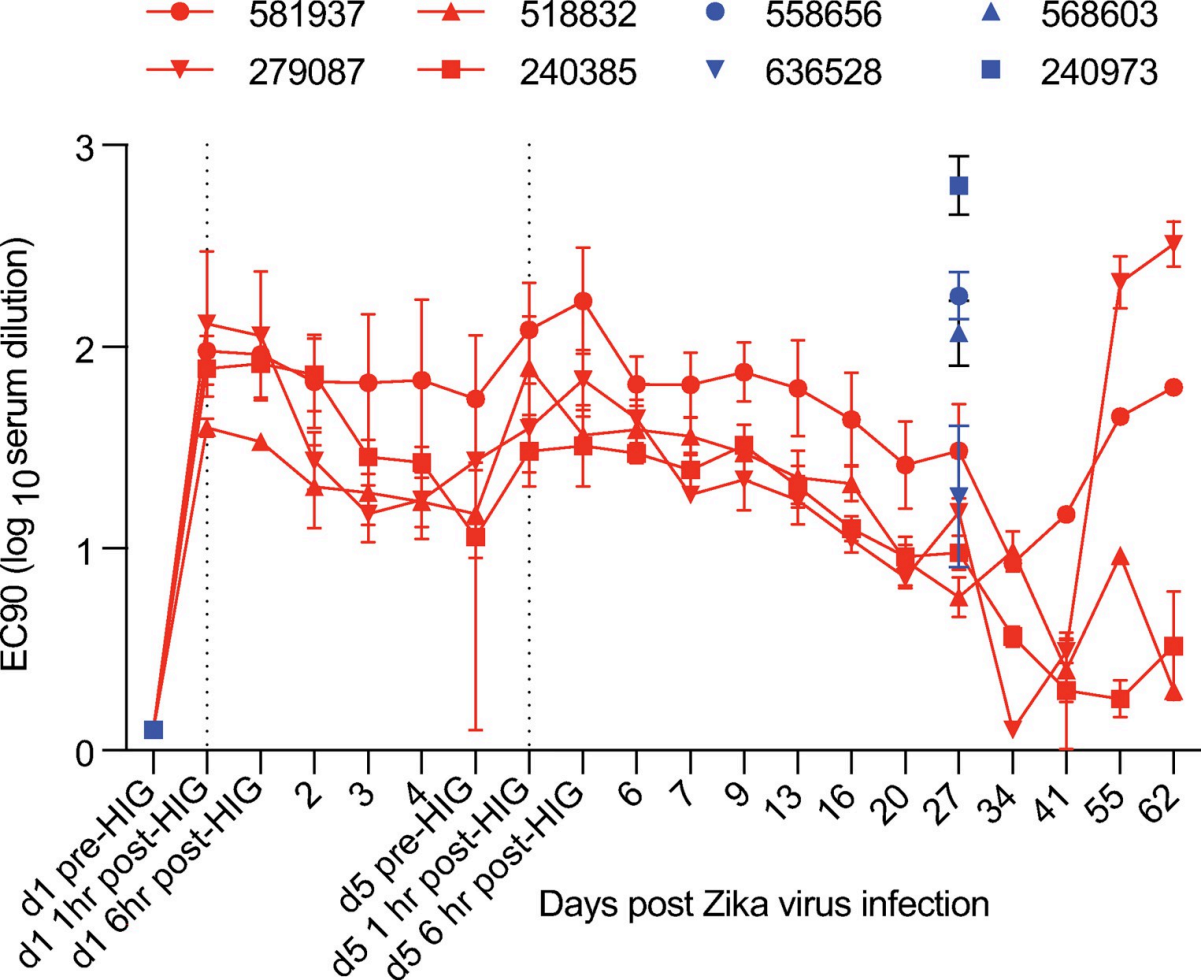
Plaque reduction neutralizing antibody titers (90%) of ZIKV-IG and placebo-IG-treated animals. Longitudinal neutralizing antibody titers of ZIKV-IG-treated animals (red). Placebo-IG PRNT90 titers were measured at 0 and 27 dpi only (blue). Dotted vertical lines represent the time point 1 hour post-ZIKV-IG infusion for reference. Mean titers with 95% CI are plotted.

Neutralizing antibody titers were measured by plaque reduction neutralization test (PRNT) daily (0–7 dpi) and then at days 9, 13, 16 and weekly through 62 dpi for ZIKV-IG-treated animals. All ZIKV-IG-treated animals had detectable titers by 1-hour post HIG infusion on 1 dpi (Fig 4). Peak 90% NAb titers ranged between 1.89 and 2.23 log_10_ serum dilution. Titers waned after the second infusion through 34 dpi, at which time NAb titers increased in two of four dams (Fig 4). This increase in NAb titer corresponds approximately to the time when macaque ZIKV- specific IgG was detected by ELISA in these two animals (Fig 3B). NAb titers were measured at days 0 and 27 post-infection for the placebo-IG-treated animals (Fig 4). In the ZIKV-IG-treated dams, the average peak NAb titer after HIG infusion and before de novo NAb response (2.04 log_10_ serum dilution, Fig 4) mirrored that of the de novo NAb response in the placebo-IG group (2.09 log_10_ serum dilution, Figs 3C and 4) on 27 dpi. This indicates that the levels of infused Ig achieved in this study matched those induced naturally by infection, which are known to protect against rechallenge [19, 24].

Non-compartmental PK analysis was performed on the ZIKV-IG-treated animals using the whole virion binding ELISA data that measures all antibodies that bind to ZIKV virions. A standard with a known concentration was used for the ELISA assay that allowed a true concentration to be calculated over time for use in PK analysis. The maximum concentration of anti-ZIKV bAbs was 1.39 mg/ml. The half-life of ZIKV-IG was 153 hours (6.4 days) and was similar to that found in macaques for other infused antibodies that also do not contain modified Fc regions meant to increase serum half-life, including rhesus IgG [25–27]. The complete PK analysis is shown in Table 1.

**Table 1:** Pharmacokinetics of ZIKV-IG based on whole virion binding ELISA presented as the geometric mean and geometric standard deviation in parentheses.

PK parameter	Definition	ELISA
C_max_ (mg/mL)	Max observed concentration	1.39
		(2.15)
AUC_0-day4_ (h*mg/mL)	Partial area under the curve between doses	47.95
		(2.18)
AUC_day4-last_ (h*mg/mL)	Partial area under the curve after second dose	215.40
		(1.91)
t_1/2 _(h)	Terminal elimination half life	153.08
		(1.49)
λ_z_ (h^-1^)	First order terminal elimination rate constant	0.005
		(1.49)
CL (mg*mL/h*mg)	Total body clearance after IV administration	0.84
		(2.30)
V_z_ (mg*mL/mg)	Volume of distribution after IV administration	185.49
		(2.07)

### ZIKV infection did not alter fetal development

To assess fetal health during pregnancy, comprehensive ultrasounds were performed once weekly and fetal heart rate was monitored twice weekly. With the exception of a few single time points, heart rates fell within normal limits (between 140 and 230 bpm) for all animals throughout pregnancy (S2 Fig). Slightly elevated heart rates were not considered concerning by the veterinary staff and no fetus exhibited a drop in heart rate below the normal range, which can signal fetal distress.

**Fig 5:**
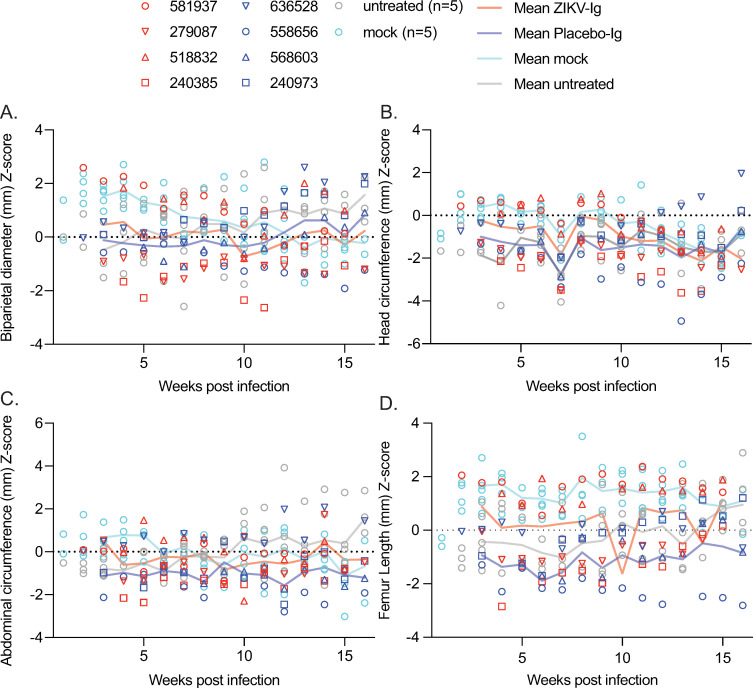
Ultrasound growth z-scores of ZIKV-IG, placebo-IG, untreated, and mock infected animals relative to normative data from CNPRC. The legend shows individual symbols for each ZIKV-IG and placebo-IG-treated animal while untreated and mock infected groups are represented by gray and turquoise open circles, respectively. Lines represent the mean z-score at each time point for each group starting at 3–4 weeks post-infection when data could be collected from all pregnancies. The dotted line represents no change from normative data set to 0. (**A**) Biparietal diameter z-scores. (**B**) Head circumference z-scores. (**C**) Abdominal circumference z-scores. (**D**) Femur length z-scores.

Measurements of head circumference (HC), biparietal diameter (BPD), femur length (FL) and abdominal circumference (AC) were taken during weekly ultrasounds. To compare growth trajectories of the different groups, z-scores were calculated for all growth outcome parameters using age-based reference data collected at CNPRC where growth of fetal macaques has been carefully documented (Fig 5) [28]. For additional comparison, growth trajectories of five untreated ZIKV-exposed animals (484880, 664184, 730627, 795784, 918724) and five mock infected control animals (020101, 044105, 044106, 044107, 044108) were also plotted (Fig 5 and Table 1). Mean z-scores for each group were compared to reference data for each week and across all weeks post-infection (wpi) using a linear mixed effects model. Overall, the HC in the ZIKV-IG, placebo-IG, and untreated groups were significantly smaller than the age-based reference data, the AC and FL were statistically smaller for the placebo-IG group, and the FL was significantly larger in the mock control group (S1-S4 Tables in S2 File). It should be noted that the age-based reference data does not account for the impact of sedation and experimental procedures used in this study on overall growth and that the mock control group is a better representation of growth expected under this study design without virus or treatment. While placebo-IG-treated fetuses had smaller than average AC, HC and FL relative to normative data, when compared to mock-infected animals the placebo-IG group only differed in FL. This significantly smaller FL is likely due to a single animal outlier in the placebo-IG group. Detailed results are presented in the S1 File.

Pairwise comparisons of z-scores between groups for each week and across all time points were also performed for each of the four growth parameters (S5 Table in S2 File). Overall, the placebo-IG and ZIKV-IG groups did not differ from each other and only differed significantly from mock-infected dams in FL. Importantly, neither group differed significantly from each other or mock infected animals in HC or BPD. Full data is presented in S5 and S6 Tables in S2 File and a detailed description of these results are presented in S1 File.

### Infant outcomes of ZIKV-IG and placebo-IG treated animals were similar

All pregnancies from both ZIKV-IG and placebo-IG-treated groups proceeded to the scheduled experimental term cesarean section time point with no pregnancy losses. Serum was collected from the fetuses and all tested negative for ZIKV IgM antibodies by ELISA. All fetuses were euthanized and measurements of at-birth HC, BPD, femur length, fetal length, fetal weight, placental diameters, and placental weights were recorded (S7 Table in S2 File). A subset of these parameters available from the mock and untreated groups were compared to the ZIKV-IG and placebo-IG groups. Fetuses from dams treated with ZIKV-IG weighed significantly more at birth than fetuses from dams treated with placebo-IG (p = 0.02) (S8 Table in S2 File). Fetuses from ZIKV-IG-treated dams did not differ from placebo-IG-treated animals in FP ratio, BPD or HC. ZIKV-IG-treated animals also weighed more than mock-infected untreated dams (p = 0.04) and untreated ZIKV-infected dams (p = 0.02) (S9 Table in S2 File). Despite a higher birthweight, fetuses from ZIKV-IG-treated dams had significantly smaller BPD and HC than untreated ZIKV-infected dams (p = 0.02, p = 0.02, respectively), but were not different from placebo-IG-treated animals (S9 Table in S2 File). Animals were also assessed for microcephaly, overlapping sutures, prominent occipital bones, redundant scalp skin, ventriculomegaly, cerebellar hypoplasia, clubfoot, microphthalmia, coloboma, and joint contractures/arthryogryposis. No gross malformations were noted in any infant.

### Fetal pathology was similar between ZIKV-IG and placebo-IG groups

All infants had extensive post-mortem examinations with tissue collection at the time of birth. Approximately 60 organs and tissues from the infant, maternal/fetal interface, and dam were tested for ZIKV by qRT-PCR (S10 Table in S2 File). All tissues tested negative for ZIKV vRNA. Placental tissues from two ZIKV-IG (279087, 518832) and two placebo-IG-treated (636528, 558656) dams were tested for ZIKV vRNA by in situ hybridization and were also negative for ZIKV RNA. Histologic findings from fetal tissues ranged from no significant histologic lesions to mild inflammation in both the ZIKV-IG and placebo-IG-treated groups (Table 2). Fetal lesions were not consistently different between groups. One eye from each of the fetuses in the ZIKV-IG and placebo-IG-treated groups was submitted to the Comparative Ocular Pathology Laboratory of Wisconsin for characterization. All fetuses had normal newborn macaque ocular morphology.

**Table 2:** Fetal pathology-morphologic diagnoses.

Dam ID	Treatment	Fetal morphologic diagnoses
581937	ZIKV-IG	No significant histologic lesions.
279087	ZIKV-IG	Mild neutrophilic otitis media, minimal neutrophilic lymphadenitis (parotid lymph node, peri-esophageal lymph node, submandibular lymph node, Inguinal lymph node, axillary lymph node, tracheobronchial lymph node, & mesenteric lymph node)
518832	ZIKV-IG	Minimal to mild neutrophilic lymphadenitis (axillary lymph node, inguinal lymph node, mesenteric lymph node, pancreatoduocenal lymph node, & submandibular lymph node).
240385	ZIKV-IG	Minimal multifocal cerebral cortical mineralization, mild neutrophilic lymphadenitis (mesenteric lymph node).
636528	Placebo-IG	Minimal to moderate neutrophilic lymphadenitis (axillary lymph node, tracheobronchial lymph node, & submandibular lymph node), mild multifocal cerebral cortical and cerebral vascular mineralization, mild multifocal periportal neutrophilic hepatitis.
558656	Placebo-IG	No significant histologic lesions.
568603	Placebo-IG	Minimal to mild neutrophilic lymphadenitis (inguinal lymph node), mild to moderate multifocal lymphocytic abdominal dermatitis, moderate multifocal lymphoplasmacytic, neutrophilic, and histiocytic abdominal panniculitis and steatitis.
240973	Placebo-IG	Mild multifocal neutrophilic lymphadenitis (axillary lymph node).

### Placental pathology was similar between ZIKV-IG and placebo-IG groups

A section cut through the entire center of each placental disc was evaluated for histopathology. Significant placental pathology was identified in all ZIKV-IG and placebo-IG animals (S11 Table in S2 File). These included regions of deciduitis, fibrinoid necrosis, and infarctions. Several cases had histologic evidence of maternal vascular malperfusion. To further understand the magnitude of these findings in the context of other placentas from control groups, placental pathology was independently scored by two pathologists for 21 features known to cause placental dysfunction, with comparisons between the two treatment groups, mock-infected animals and untreated ZIKV-exposed animals (only for % transmural infarction) (S12 Table in S2 File). Animals in the ZIKV-IG and placebo-IG groups underwent three MRIs during pregnancy for another study, which involved treatment with ferumoxytol as a contrast reagent to measure blood flow through the placenta. As an additional control group for placental pathology, dams with ZIKV infection that were also treated with ferumoxytol and sedated for MRI, but not treated with HIG, were assessed (MRI group). Full histopathology scores from two independent pathologists are shown in S13 Table in S2 File while pairwise comparisons of the mean scores (S14 Table in S2 File) are shown in S15 Table in S2 File. Pairwise comparison of each feature between these 5 groups did not show a significant difference in pathology in any one group across multiple features. Additionally, no group differed from more than one other group for a single feature (S15 Table in S2 File). No significant differences were found between the ZIKV-IG and placebo-IG groups. Compared to the MRI group, ZIKV-IG + placebo-IG animals grouped together had increased diffuse perivillous fibrin in both placental discs (S16 Table in S2 File). Additional analysis between HIG treated and MRI groups are provided in S1 File and S16-S18 Tables in S2 File.

### No anti-human antibodies were detected in the IgG-treated dams

Since the HIG Abs used in the rhesus macaques in this study were isolated from human donors, we tested whether the dams mounted a systemic anti-human IgG antibody response against the infused Ig. An ELISA assay was developed as described in the methods section (S3 Fig). No animals had levels of macaque anti-human Abs above the negative control serum from untreated animals.

## Discussion

In this study, ZIKV-IG significantly reduced the duration and peak plasma viremia in rhesus macaque dams treated one day after ZIKV infection. The control of viremia was potent throughout the time the human antibodies were detectable in the blood. As the ZIKV-IG titer waned, one animal had viral recrudescence at 41 dpi, suggesting that ZIKV-IG does not always clear viral replication from all compartments in the dams. Additional work is necessary to understand how long treatment might be required in women with confirmed ZIKV infection to prevent recrudescence during pregnancy, which might be throughout the duration of pregnancy. Two of four of the ZIKV-IG-treated dams developed a de novo NAb response, which could indicate viral replication in a non-blood compartment site in these animals once infused antibody levels waned. Alternatively, this could also represent a delayed antibody response to the initial replication prior to treatment. Unfortunately, we did not collect peripheral blood mononuclear cells, amniotic fluid, or biopsies to look for virus in lymph nodes or other compartments in this study. Therefore, we cannot rule out that virus entered the fetal compartment even if it was controlled in the plasma, though we also have no indication of ZIKV-induced damage in the fetuses. While we considered these procedures, choosing the correct timing for biopsies is difficult to catch viral replication and amniocentesis poses risks to the pregnancy such as introduction of bacteria into the fetal compartment that could lead to miscarriage, or direct transmission of ZIKV into the fetal compartment as the needle passes through tissues into the amniotic sac. The possibility that amniocentesis might bypass natural vertical transmission that may be controlled by the therapeutic represents an avoided but possible confounding factor for therapeutic studies. Two other dams did not develop a NAb response and never had detectable virus after treatment suggesting ZIKV-IG may have eradicated ZIKV in at least half the animals. Altogether, these data suggest that ZIKV-IG can quickly control ZIKV infection in pregnancy and has significant potential as a potent treatment or prophylaxis to reduce the risk of congenital Zika syndrome. Indeed, vaccine-induced reduction of duration and peak maternal viremia was shown to reduce CZS outcomes in another NHP model [9].

A careful assessment of the health of the dam and fetuses throughout pregnancy suggest that the fetuses grew similarly between the ZIKV-IG and placebo-IG animals and to mock-infected animals and did not exhibit signs of fetal distress. In this study, ZIKV-exposed fetuses had a smaller HC than mock-infected animals, when measured in utero, but ZIKV-IG and placebo-IG-treated animals did not. Since we saw the same impact in both ZIKV-IG and placebo-IG groups, we cannot conclude that ZIKV-IG specifically altered this outcome. Since there were no ZIKV-associated adverse outcomes exhibited in the placebo-IG group, no conclusions can be drawn on the impact of ZIKV-IG on fetal outcomes. However, it is important to note that ZIKV-IG appeared safe in these pregnancies, without adverse fetal effects or impact on growth of the fetuses relative to mock-infected animals.

Overall growth and development of a fetus is often dependent on placental function. Pathological analysis of the ZIKV-IG and placebo-IG groups showed evidence of placental pathology including deciduitis, fibrinoid necrosis, and infarctions. However, comparison of pathology scores between experimental groups, including ZIKV-IG, placebo-IG, mock-infected, and ZIKV-infected, indicated no consistent statistical difference. Combined with the fact that all infants appeared normal in size and gross morphology with minimal significant histopathology, suggests that the placental pathologies noted histologically were insufficient to cause enough placental dysfunction to impact the growth and development of the fetus. One pathological finding of increased perivillous fibrin in Ig-treated dams (ZIKV-IG and placebo-IG groups combined) was noted relative to the MRI group. Increased perivillous fibrin can occur as a result of several different possible causes including direct injury to the syncytiotrophoblast, diminished local perfusion to the intervillous space such as from maternal vascular malperfusion, or idiopathic such as in massive perivillous fibrin deposition [29, 30]. One potential cause for increased perivillous fibrin in both Ig-treated groups compared to the mock controls is direct injury to the syncytiotrophoblast from nonspecific antibody binding to the syncytiotrophoblast that could be activating complement. Interestingly, human IVIG is used to treat several disorders in pregnancy, and does not seem to lead to direct syncytiotrophoblast injury in those instances, suggesting that the nonspecific injury to the rhesus macaque villi may be a species-specific effect of the administration of human IgG [31, 32]. There has been some evidence of increased fetal growth restriction and perhaps pre-term birth in a recently tested HIG product to prevent congenital CMV; therefore it is important to continue to assess safety of these products during pregnancy [33]. Future studies, planned to continue to test the efficacy of ZIKV-IG in rhesus macaques, will be performed with macaque-derived antibodies to avoid any cross-species effects. Overall, the lack of adverse fetal outcomes in these Ig-treated animals suggests that the benefit of these antibodies to reduce maternal viral load outweighed any risk of using HIG during pregnancy.

There are still no therapeutics or vaccines that are FDA approved to combat Zika virus either for pregnant or non-pregnant individuals. The advantage of HIG over a vaccine is that it immediately controls ZIKV infection and can be used before or after exposure while vaccines would need to be used weeks before a possible exposure to prevent infection. The data presented here is a promising first step in considering the use of ZIKV-IG in pregnant women exposed to ZIKV. The primary caveat to this study is that there were no adverse fetal outcomes in the placebo-IG group making it impossible to directly assess efficacy of ZIKV-IG against CZS. Nor was virus detectable in either treatment group in maternal/fetal interface or fetal tissues as an alternative measureable outcome. One caveat to the data presented from the placenta is that we only sampled 3 sections from each placenta and may have missed virus present in other sections of the placenta. However, in previously published untreated animals infected with the same virus, dose, and time of gestation as animals in this study, virus was not consistently detected in the placenta even when sampling each cotyledon [23]. Therefore, the results found in this study mirror results from other studies using the same infection model. It is also possible that ZIKV-PR does not vertically transmit as well as other ZIKV strains. While initial evidence from multiple studies across different primate centers suggested that about 1 in 4 pregnancies ended with adverse fetal outcomes, the data generated since this study was initiated suggests that the specific model used here with ZIKV-PR infection at GD45 generates adverse fetal outcomes in approximately 1 in 7 pregnancies, similar to what is established in humans [34, 35]. Therefore, we did not have sufficient animal numbers to see a clear difference in fetal outcomes. Since this study, our group is developing an alternative model of ZIKV infection in rhesus macaques that produces higher rates of adverse fetal outcomes. The data from this study suggest that ZIKV-IG warrants further testing in this updated model where pregnancy outcome and impact on maternal viral load can be studied together. We also plan to increase group sizes. Since one day post-infection is rarely a feasible time point for treatment, this future work will also begin to define how long after infection it is possible to treat with ZIKV-IG and still reduce risk or severity of CZS.

Antibody-dependent enhancement (ADE) associated with flavivirus infections is a known phenomenon that occurs when antibodies generated after infection with one dengue virus (DENV) serotype causes enhanced disease after infection with a different DENV serotype. Because ZIKV is highly related to dengue virus, ADE has been a concern that requires consideration with anti-ZIKV antibody treatments and vaccines. One potential concern with treating pregnant women with ZIKV-IG is that it could lead to enhanced ZIKV or DENV infection for a period of time after receiving the antibody treatment and/or prophylaxis. While ADE can lead to enhanced disease after DENV infection, there is little evidence of enhanced disease after ZIKV infection in either humans or nonhuman primates, though there are reports in mouse models [36, 37]. In this study, we did not see evidence of enhanced ZIKV infection in our treated dams. More probable is the possibility that ZIKV-IG treatment could increase the risk of severe dengue disease in subsequent DENV infection given the established propensity for ADE with DENV and recent epidemiological evidence that prior ZIKV infection enhances this risk in humans [38]. Antibodies against ZIKV can cross-react with DENV in PRNT assays and can enhance infection in cell culture-based ADE assays [39]. There could be a small window of time, which would need to be established, where infused anti-ZIKV antibody titers wane and enhancement of DENV could occur if the individual was infected with DENV in that window of time. However, as an infused antibody, it is expected that this level would not persist long and may make this option more appealing than a vaccine that might elicit longer-lasting antibody responses. Since ADE occurs within a very specific range of antibody titers (DENV iELISA antibody titer from 1:21 to 1:80) [40, 41], the benefits of preventing CZS with antibody prophylaxis or treatment likely far outweighs the possible risk of DENV infection at just the right time to cause enhanced DENV disease. Nonetheless, understanding ADE associated with ZIKV-specific immunotherapy needs to be thoroughly evaluated for promising candidates like ZIKV-IG.

The ZIKV-IG product tested here was also used in a phase I clinical trial to assess safety in humans. Clinically, ZIKV-IG could be highly effective as a prophylaxis. Since treatment at one day post-infection dramatically reduced plasma viral load, it can be hypothesized that treatment shortly before infection would likely prevent detectable viremia in the infected individual. When used during pregnancy, when ZIKV infection causes the highest disease burden, treatment would be expected to minimize vertical transmission and reduce incidence of CZS among infected women. If a prophylaxis such as ZIKV-IG were available, it may warrant more frequent pregnancy testing in ZIKV endemic areas where ZIKV-IG could be administered immediately after confirmation of pregnancy to protect from first trimester infections. ZIKV infections have remained low over the past few years in regions with the mosquitoes that carry the virus, making it very difficult to assess efficacy of treatments or vaccines in humans. Pregnant women are often left out of vaccine trials so even if a vaccine was shown to be effective, it may not be an option during pregnancy [42]. On the other hand, HIG has been approved for use during pregnancy to prevent adverse pregnancy outcomes for other pathogens and conditions [12, 13]. The data presented here supports further characterization of the impact of this product to prevent adverse fetal outcomes associated with ZIKV infection as both a prophylaxis and treatment. These results, therefore, support the continued development of HIG as a medical countermeasure that could be stockpiled in advance of future ZIKV outbreaks to protect pregnant women.

## Materials and methods

### Study design

This study was designed to test the hypothesis that immunoglobulin isolated from individuals previously infected with ZIKV can reduce maternal viral load and prevent congenital Zika syndrome when used at one- and five- days post-infection relative to placebo treated animals. Eight pregnant, Indian-origin female rhesus macaques (*Macaca mulatta*) were infected at approximately gestational day (GD) 45 with Zika virus/H.sapiens-tc/PUR/2015/PRVABC59-v3c2 (ZIKV-PR). Four animals were chosen for each group based on data showing that one in four rhesus macaque pregnancies showed evidence of adverse fetal outcomes from previous studies [34]. Animals were randomly assigned to either a treatment or placebo group. At one- and five-days post infection (dpi) animals received 50mg/kg of either ZIKV-specific human immune globulin (ZIKV-IG) or non-specific human immune globulin (placebo-IG) administered intravenously (IV) at a rate of approximately 1 mL/minute. All pregnancies were monitored weekly by ultrasound, and samples were collected for viral load and antibody quantification at predefined times throughout the study. At approximately GD 155, fetuses were delivered by cesarean section (c-section), euthanized, and a fetal necropsy was performed to assess fetal and maternal-fetal interface tissues for ZIKV RNA and pathology. Due to small animal numbers, outliers were included in analyses. To further evaluate the impact of ZIKV-IG and placebo-IG on fetal growth and pathology, up to six mock infected animals and nine untreated ZIKV-exposed animals from other studies were included in the analyses (Fig 1).

### Care and use of macaques

The macaques used in this study were cared for by the staff at the Wisconsin National Primate Research Center (WNPRC) in accordance with recommendations of the Weatherall report and the principles described in the National Research Council’s Guide for the Care and Use of Laboratory Animals [43]. The University of Wisconsin—Madison, College of Letters and Science and Vice Chancellor for Research and Graduate Education Centers Institutional Animal Care and Use Committee approved the nonhuman primate research covered under protocol number G005401. The University of Wisconsin—Madison Institutional Biosafety Committee approved this work under protocol number B00000117. All animals were housed in enclosures with required floor space and fed using a nutritional plan based on recommendations published by the National Research Council. Animals were fed a fixed formula, extruded dry diet with adequate carbohydrate, energy, fat, fiber, mineral, protein, and vitamin content. Macaque dry diets were supplemented with fruits, vegetables, and other edible objects (e.g., nuts, cereals, seed mixtures, yogurt, peanut butter, popcorn, marshmallows, etc.) to provide variety to the diet and to inspire species-specific behaviors such as foraging. To further promote psychological well-being, animals were provided with food enrichment, structural enrichment, and/or manipulanda. Environmental enrichment objects were selected to minimize chances of pathogen transmission from one animal to another and from animals to care staff. While on study, all animals were evaluated by trained animal care staff at least twice each day for signs of pain, distress, and illness by observing appetite, stool quality, activity level, physical condition. Animals exhibiting abnormal presentation for any of these clinical parameters were provided appropriate care by attending veterinarians. Prior to all minor/brief experimental procedures, macaques were sedated using ketamine anesthesia and monitored regularly until fully recovered from anesthesia. At the end of the study all animals were released back to their home colony.

### Viral infection

Zika virus/H.sapiens-tc/PUR/2015/PRVABC59 (ZIKV-PR) was originally isolated from a traveller to Puerto Rico, propagated with three rounds of amplification on African green monkey kidney cells (Vero cells, ATCC #CCL-81), obtained from Brandy Russell (CDC, Ft. Collins, CO). Virus was prepared for this and other studies by inoculation onto *Aedes albopictus* mosquito cells (C6/36 cell, ATCC #CRL-1660) with two rounds of amplification [44]. Macaques were subcutaneously inoculated over the cranial dorsum with 1mL of 1x10^4^ PFU of the resulting stock. Back titration was performed with the inoculum used for the first- and last-infected animals and fell within the 0.5 log error rate of the plaque assay for both infections.

### Temperature and body weight measurement

Rectal temperatures and body weights were collected throughout the study. WNPRC veterinary staff were consulted in determining whether elevation of an individual animal’s body temperature beyond reference ranges was clinically significant.

### ZIKV RNA isolation and quantification by qRT-PCR

ZIKV RNA was isolated from plasma and tissues and quantitated by qRT-PCR as previously described [24, 45]. Plasma was isolated from EDTA-treated whole blood by centrifugation at 1400 x rcf for 15 minutes followed by transfer to a sterile tube and a second centrifugation at 670 x rcf for 8 minutes. Serum was obtained from clot activator tubes by centrifugation at 670 x rcf for 8 minutes. Viral RNA (vRNA) was extracted from 300uL plasma using a Maxwell 16 MDx instrument (Promega, Madison, WI) as previously described [24, 45]. Tissue samples collected at c-section and necropsy were stored in RNAlater at 4°C for approximately 24 hours prior to removal of excess RNAlater and freezing at -80°C. RNA was recovered from tissue samples using a modification of the method described by Hansen et al. [46]. Briefly, up to 200 mg of tissue was disrupted in TRIzol Reagent (Thermo Fisher Scientific, Waltham, MA) with 2 x 5 mm stainless steel beads using a TissueLyser (Qiagen, Germantown, MD) for 3 minutes at 25 r/s twice. Following homogenization, samples in TRIzol were separated using Bromo-chloro-propane (Sigma, St. Louis, MO). The aqueous phase was collected into a new tube and glycogen was added as a carrier. The samples were washed in isopropanol and ethanol precipitated overnight at -20 C. RNA was fully re-suspended in 5 mM Tris pH 8.0. RNA concentrations from plasma and tissue samples were determined by interpolation onto an internal standard curve of seven ten-fold serial dilutions of a synthetic ZIKV RNA segment based on ZIKV-FP. The limit of quantification of this assay is 100 copies vRNA/mL plasma or serum [24, 45].

### Plaque reduction neutralization tests (PRNT_90_)

Titers of ZIKV neutralizing antibodies were determined from macaque serum samples using PRNT on Vero cells (ATCC #CCL-81) with a cutoff value of 90% (PRNT_90_) against ZIKV-PR [45]. Neutralization curves were generated in GraphPad Prism (San Diego, CA) and the resulting data were analyzed by nonlinear regression to estimate the dilution of serum required to inhibit 90% Vero cell culture infection.

### Viral quantification by plaque assay

Plaque assays were performed on Vero cells (ATCC #CCL-81) to quantitate and back-titrate ZIKV stocks. Assays were performed in duplicate. Cells were inoculated with 0.1 mL aliquots from serial 10-fold dilutions of ZIKV in growth media as described previously [44, 45].

### Ultrasonography and fetal monitoring

Ultrasounds and fetal doppler were conducted weekly to observe the health of the fetus and to obtain measurements including fetal femur length (FL), biparietal diameter (BPD), head circumference (HC), abdominal circumference (AC) and heart rate as previously described [47, 48]. Mean growth measurements were then plotted against mean measurements and standard deviations from specific gestational days collected from rhesus macaques by Tarantal et al. [28].

### Human immune globulin (HIG) manufacture

Zika virus human immune globulin (ZIKV-IG) was purified from human donors using plasma collected from US FDA-licensed plasma centers screened for high antibody reactive to ZIKV [49]. The manufacturing process includes purification by lipoprotein removal and anion exchange chromatography [50]. It also includes processes to increase product safety by reducing the risk of transmission of several viruses including human immunodeficiency virus (HIV), hepatitis B virus (HBV) and hepatitis C virus (HCV) by removal of lipid-enveloped and non-enveloped viruses based on size as well as inactivation of lipid-enveloped viruses by solvent detergent (SD) treatment. The SD treatment was removed by reverse-phase column chromatography.

The ZIKV-IG product (lot PD_740_ZKP_16_001_003_ER_v1) used in these studies contained a protein concentration of 54 mg/mL (>98.9% human IgG) with a microneutralization titer of 12245 based on a xCELLigence real-time cell analysis (RTCA, ACEA Biosciences Inc.) [49].

Placebo human Ig (placebo-IG) was purified through the same manufacturing process using plasma without antibody reactive to ZIKV. Placebo Ig (PD _740_ZKP _16_001_007) contained a protein concentration of 59 mg/mL (>99.5% human IgG) with undetectable ZIKV neutralization titer.

### Histopathology

Tissues collected for histopathology were fixed in 4% PFA, embedded in paraffin, sectioned as previously described [47, 48], and stained with hematoxylin and eosin. Veterinary pathologists were blinded to the vRNA status of tissues and determined microscopic diagnosis as previously described. Placental pathology from a center cut of each disc was scored for 21 features known to cause placental dysfunction by two pathologists independently. See S12 Table in S2 File for the features that were scored and the scoring algorithm for each feature.

### In situ hybridization

Necropsy tissues were fixed in 4% PFA, alcohol processed, paraffin embedded, and then prepared for in situ hybridization (ISH) as described previously [48].

### ZIKV IgM ELISA

Fetal serum was assessed for the presence of ZIKV-specific IgM by ELISA (Euroimmun, Lübeck, Germany) except for 726723 for which fetal plasma was used because serum was not collected. Serum collected from an animal (480311) during acute ZIKV infection served as an internal positive control. All samples were tested in duplicate and in 1:100 dilution, per manufacturer recommendations. Optical density was measured at 450 nm and 600 nm within 30 minutes of stop solution addition on the Glomax Multi-Detection System (Promega, Madison, WI). Extinction values were calculated using an equation provided by Euroimmun. Values of < 0.8, 0.8–1.1, and > 1.1 were classified as negative, borderline, and positive, respectively.

### Whole virion binding ELISA

The whole virion binding ELISA was performed as described previously with the addition of a second whole virion binding ELISA that included the use of a different secondary antibody: horseradish peroxidase (HRP)-conjugated goat anti-human IgG antibody (Southern Biotech, Birmingham, AL) to track the titer of human-derived Ig used to treat the animals [37]. Optical density (OD) was detected at 450 nm on a Victor X Multilabel plate reader (PerkinElmer, Waltham, MA). The limit of detection was defined as an OD value of the 1:12.5 dilution greater than three times the background OD of ZIKV naive macaque plasma. The log_10_ 50% effective dilutions (ED50) were defined as the plasma dilutions at which there was a 50% decline in the maximum IgG virion binding based on the OD. Whole virion IgG binding responses were examined across all time points (0–62 dpi).

### Pharmacokinetics analysis

Whole ZIKV virion binding ELISA data from days 0–62 post infection were used for pharmacokinetic (PK) analysis [37]. Data were analyzed by standard non-compartmental methods (i.e., trapezoidal method) using SAS 9.4 Enterprise Guide (EG) (SAS Institute Inc., Cary, NC, USA) and the following PK parameters were derived: AUC0-4 and AUC0-t by linear trapezoidal rule (linear up/log down), Cmax (mathematic derived), λz, t1/2, CL, and Vz.

### Anti-human macaque antibody detection ELISA

An ELISA was designed to detect the presence of ant-human macaque antibodies in serum samples from both treatment and placebo groups. Briefly, ZIKV-IG or placebo-IG was coated to a 96-well maximum binding plate (Invitrogen IgG (Total) Human Uncoated ELISA Kit, cat. # 50-112-9727) in a coating buffer at 2μg/ml and allowed to incubate at 4°C overnight. The recombinant human IgG standard from the Invitrogen ELISA kit was set up in 1:2 dilutions per the manufacturer’s protocol in duplicate on each plate tested. The plate was then washed in an ELISA washing buffer (Thermo Fisher Scientific, Waltham, MA) and treated with blocking buffer (Bio-Rad Laboratories, Hercules, Ca) was added to incubate overnight at 4°C. The plate was then washed three times using the same washing buffer and serum was diluted 1:100, 1:1000 and 1:10,000 in the blocking buffer and added to the plate to incubate at room temperature for 2hrs. After washing a third time, horseradish peroxidase (HRP)-conjugated mouse anti-macaque IgG antibody (Southern Biotech, Birmingham, AL) was incubated in each well for 1hr at room temperature before being washed. To ensure that the wells were coated, an HRP-conjugated goat-anti-human IgG polyclonal secondary antibody was applied to two wells incubated without serum in the previous step (Southern Biotech, Birmingham, AL) (S3 Fig). TMBE substrate (Thermo Fisher Scientific, Waltham, MA) was placed in each well and incubated at room temperature for 15 mins. to detect macaque or human IgG. The plate was then read at OD 400nm and 600nm. Several controls were run to ensure that the antibodies would not non-specifically bind to the human Abs on the plate or to non-specific antibodies present in the serum. Without serum, the anti-macaque Ab did not bind to the human IgG products bound to the plate and would not result in false positive results. Addition of serum from untreated or ZIKV-IG treated animals at 1 dpi, which are expected to not have anti-human antibodies yet showed some cross reactivity with the anti-macaque antibodies (S3 Fig). This indicates that it is necessary to use an untreated control on each plate to subtract background binding of Abs in macaque serum detected with the anti-macaque Ab. Addition of serum did not prevent the anti-human Ab from binding to the human Abs on the plate. Lastly, serum from ZIKV+ animals containing anti-ZIKV Abs also did not interfere with the anti-human Abs binding to the human Abs on the plate and there was some background binding of the anti-macaque Ab, but less than that found in the serum on an untreated and uninfected animal. Overall, the background binding of the anti-macaque Ab was about half the absorbance found in the positive control of anti-human antibodies binding to the ZIKV-IG applied to the plate. In addition, the anti-macaque IgG antibodies were tested in a sandwich ELISA on a commercial anti-Zika ELISA plate coated with NS1 protein with an animal exposed to Zika virus with a positive NAb titer at the time point tested. As shown in S3 Fig. (blue bars), the anti-macaque Ab strongly detected the macaque anti-Zika antibodies in this animal and would be expected to bind to macaque anti-human Abs.

### Statistical analysis

Longitudinal plasma vRNA loads were compared between ZIKV-IG-treated animals, placebo-IG-treated animals, and untreated control animals infected with ZIKV-PR over time by calculating the area under the curve (AUC) for each animal’s plasma vRNA trajectory using the trapezoid rule. The AUCs were compared between groups using the nonparametric Kruskal-Wallis test. Each treatment group was compared to the control group using Dunnett’s test to control the type I error (<0.05).

The duration of detection of vRNA in plasma for each animal was examined using two different definitions: a) duration defined as the last dpi a positive vRNA load was detected by qRT-PCR and b) duration defined as the total number of positive detections from the time points tested for each animal. For both of these datasets, the nonparametric Kruskal-Wallis test was used to compare ZIKV-IG-treated, placebo-IG-treated, and untreated control animals. Comparisons of each treatment group to the control group was conducted using Dunnett’s test. All vRNA load analyses were done in R Studio (v.1.1.383) [51].

For in utero growth comparisons between groups, age standardized z-scores were calculated for all growth outcome parameters using age (week post infection) reference data. A z-score of 0, for example, corresponds to the median of the reference population while a z-score of 1.64 corresponds to the upper 95^th^ percentile. A linear mixed effects model with animal specific random effects and an autoregressive correlation structure was used to evaluate z-scores within groups and to compare z-scores between groups at each week post infection and across all weeks post infection. Gender, dam age and weight were included as covariates. Least squares adjusted means are reported along with the corresponding 95% confidence intervals. Tukey’s Honestly Significant Difference (HSD) method was utilized to control the type I error when conducting multiple comparisons between groups. Outliers were assessed but included in all analyses.

To assess placental pathology between groups, two independent pathologists scored each placenta for each category (as described in the histopathology methods section). The inter-rater variability between pathologist’s ratings for each feature was assessed using the intra-class correlation (ICC) for features measured on a quantitative scale and the Kappa index for features measured on a binary scale (present/absent). Since the inter-rater variability was good to excellent (ICC or Kappa >0.75) for the majority of features (S19 Table in S2 File), outcome scores were calculated based on averaging the feature scores between the two pathologists. Each placental disc was evaluated separately. Outcome scores were summarized in terms of median and interquartile ranges for each group and placental disc. Comparisons between groups were conducted using the nonparametric Wilcoxon rank sum test.

For in utero growth and placental pathology statistics, all reported p-values are two-sided and P<0.05 was used to define statistical significance. Statistical analyses were conducted using SAS software (SAS Institute, Cary NC), versions 9.4.

Birthweight and fetal-placental-ratio were analyzed using an analysis of covariance model. BPD and HC were analyzed using a multivariate analysis of covariance model with two dependent variables. Analysis was performed in R Studio (v.1.1.383) [51].

## Supporting information

S1 Fig Longitudinal measurements of maternal temperature and weight for ZIKV-IG- and placebo-IG-treated animals. (TIF)

S2 Fig Longitudinal fetal heart rate of ZIKV-IG- and placebo-IG-treated animals. (TIF)

S3 Fig Testing controls for the macaque anti-human ELISA assay. (TIF)

S1 File Extended results section. (DOCX)

S2 File Supplementary data presented in tables. (XLSX)
