# Supplementary figures and images for: Human immune globulin treatment controls Zika viremia in pregnant rhesus macaques
Source: PLoS One. 2022 Jul 14;17(7):e0266664. doi: 10.1371/journal.pone.0266664 (PMC9282477; doi:10.1371/journal.pone.0266664)

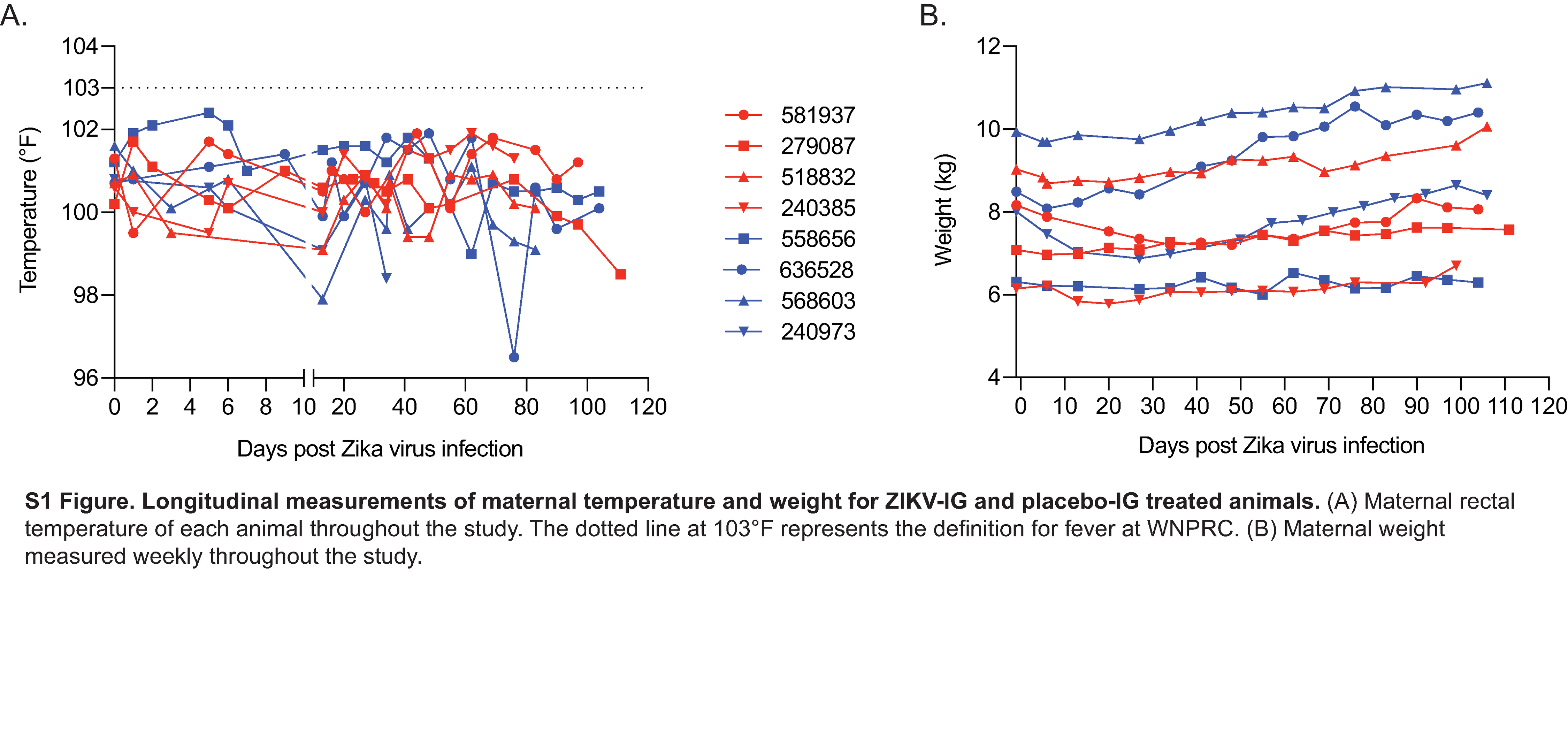

Supplement: S1 Fig — (TIF) [file pone.0266664.s001.tif]

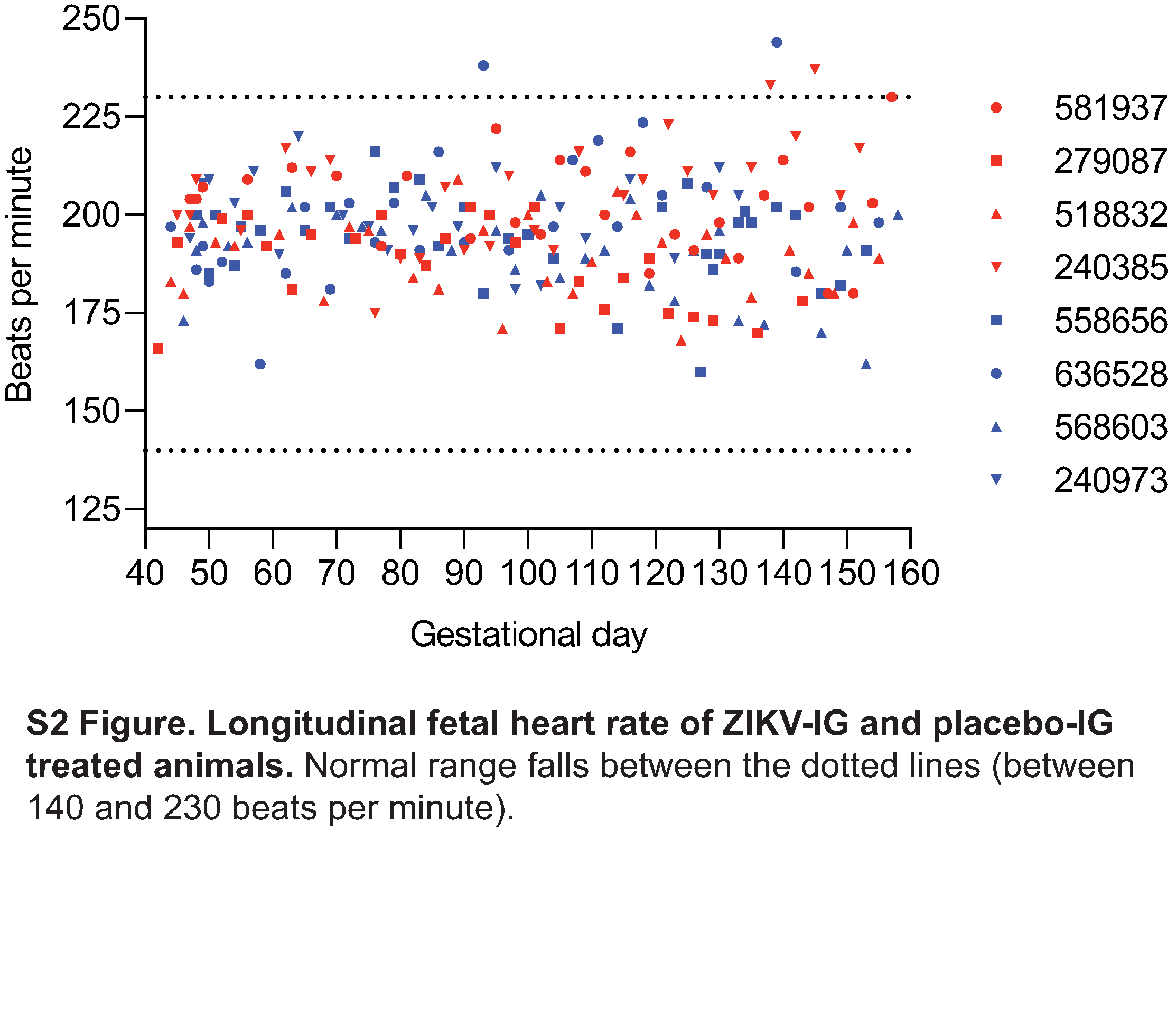

Supplement: S2 Fig — (TIF) [file pone.0266664.s002.tif]

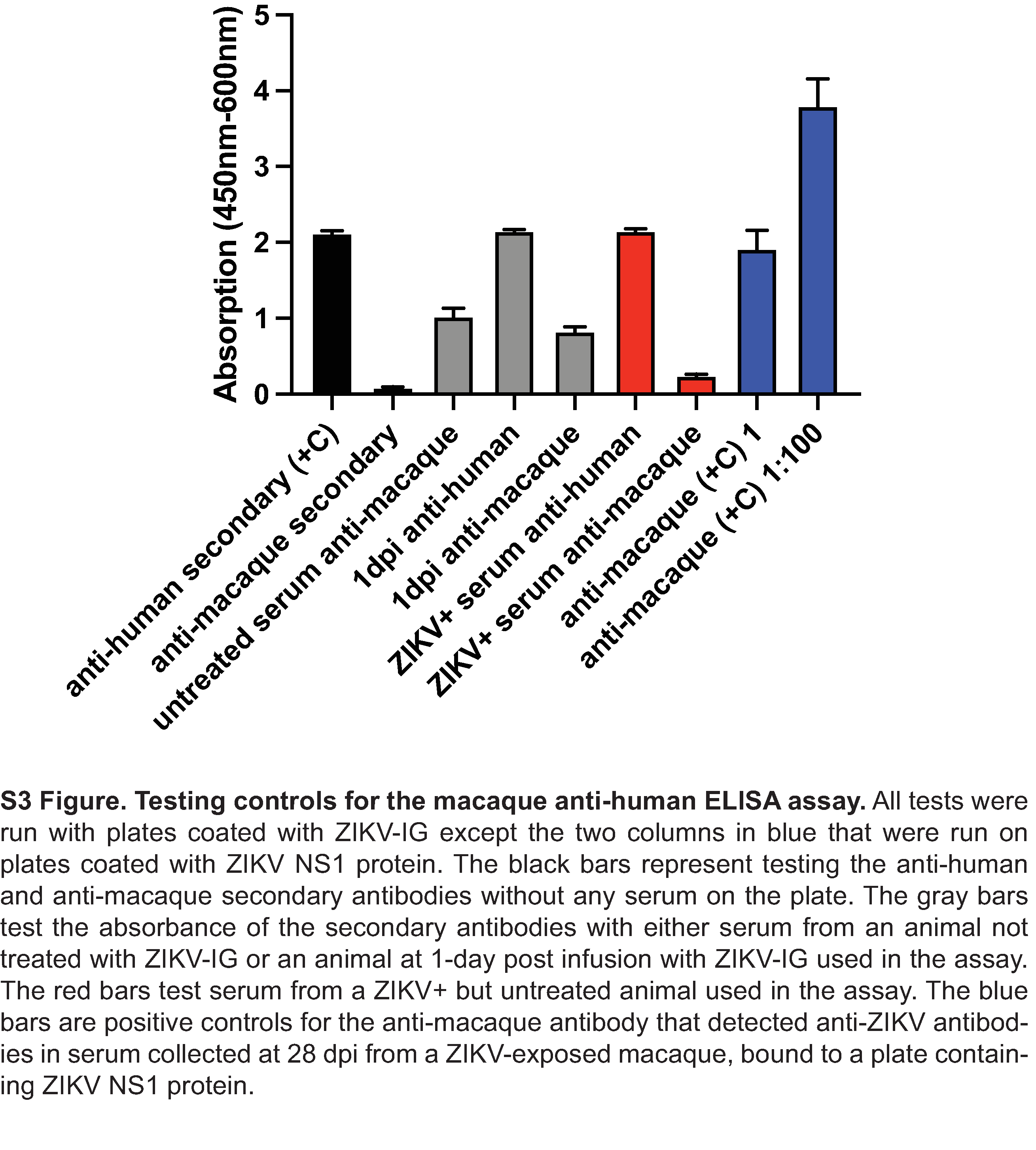

Supplement: S3 Fig — (TIF) [file pone.0266664.s003.tif]
