# Supplementary material for: Human immune globulin treatment controls Zika viremia in pregnant rhesus macaques
Source: PLoS One. 2022 Jul 14;17(7):e0266664. doi: 10.1371/journal.pone.0266664 (PMC9282477; doi:10.1371/journal.pone.0266664)
Supplement: S1 File — (DOCX) [file pone.0266664.s004.docx]

**S1 Results**

**Few clinical signs of infection and no reaction to Ig infusion in dams**

Following ZIKV infection and HIG infusion, animals were carefully monitored for clinical signs of infection and reaction to HIG infusion. All animals were prophylactically administered diphenhydramine prior to HIG infusion and no adverse reactions occurred during or after HIG infusion. One placebo-IG treated dam showed signs of a rash at the viral injection site after ZIKV infection noted at 5 dpi (240973). Overall, dams showed no other clinical signs of Zika infection including no conjunctivitis, generalized rash, fever, change in temperament, nor significant weight loss.

Temperatures were monitored at least weekly throughout the study and did not fluctuate more than three degrees from baseline for any animal, which is the range of normal fluctuation associated with animal handling and sedation. No animal exhibited a fever as defined as over 103°F at any time point tested (S1 Fig.). Average temperatures were similar between the ZIKV-IG and placebo-IG treated groups. The dam’s weights were tracked weekly throughout the study. Several animals exhibited a slight dip in weight during the first 2-3 weeks of the study, but then recovered and continued to increase in weight as expected for pregnant animals (S1 Fig.). Most animals lost less than 10% of their baseline body weight before recovering, except 581937 (12%) and 240973 (15%). Animals 581937 and 240973 exhibited increased inappetence for chow shortly after enrollment in this study, which could be due to housing changes, Zika infection, or daily sampling, and both animals received supplemental food to stabilize weight.

**In utero growth measured by ultrasound**

Measurements of head circumference (HC), biparietal diameter (BPD), femur length (FL) and abdominal circumference (AC) were taken during weekly ultrasounds. For BPD, mock infected animals were significantly higher at 3 and 4 wpi (p=0.01, p=0.01), the untreated ZIKV-exposed animals were significantly high at 16 wpi (p=0.01), but all other groups were not significantly different than the 50th percentile (z-score=0) at any time point and no groups were significantly different across all time points considered together (S1 Table). Head circumference was statistically smaller in weeks 4-10 for the untreated Zika group and placebo-IG group, and starting at week 11 the ZIKV-IG and at week 13 the mock infected groups also measured consistently smaller than the 50th percentile until week 16 when only the ZIKV-IG group was statistically smaller (S2 Table). Across all time points, only the mock infected group was not statistically smaller than the 50th percentile. Abdominal circumference measured statistically smaller than the 50th percentile in the placebo-IG group in weeks 8, 11, 12, and 16 (S3 Table). Other groups had 1 or 2 weeks when the AC measured small, but they were not consistently small. Across all time points for AC, only the placebo-IG was statistically smaller than the 50th percentile. Femur length was small across several early time points for the placebo-IG group and was consistently larger than the 50th percentile for the mock infected group across multiple time points (S4 Table). The ZIKV-IG group was statistically small only at week 10 (p=0.01) and 16, though week 16 was excluded because it was only collected in one of four animals and the value for the animal collected did not follow the course of growth observed prior to that time point and was considered an outlier. Across all time points, the placebo-IG group had a statistically smaller AC (p=0.02) than the 50th percentile and the mock control group was statistically larger (p=0.005) (S4 Table).

Pairwise comparisons between each group were also assessed. For BPD, untreated ZIKV-exposed animals differed from mock infected animals at 3-4 wpi (p= 0.02, p=0.02). At no other time point nor overall did these or the other groups differ from each other indicating that BPD growth of each of our treatment groups did not differ from mock-infected animals nor untreated ZIKV-exposed animals (S5 Table). For HC, the placebo-IG group differed from mock across multiple time points and untreated ZIKV-exposed animals differed from mock infected animals for some time points (S5 Table). However, across all time points, only the untreated ZIKV-exposed animals differed from mock infected (p=0.04). This indicates that our treated animals HC grew similarly to each other and to mock-infected animals. For AC, while there were spurious time points where groups differed from each other during gestation, only the untreated ZIKV exposed and placebo-IG groups differed significantly in AC growth across all time points with the untreated animals trending larger and placebo-IG animals trending smaller in growth (p=0.01) (S5 Table). FL was significantly different at multiple time points between the untreated ZIKV-exposed and mock infected groups as well as the placebo-IG and mock infected groups (S5 Table). Across all time points, only the placebo-Ig group was statistically different from the mock group (p=0.0147).

Lastly, to assess the impact of any HIG infusion on macaque pregnancy, we combined the ZIKV-IG and placebo-IG animals into a single group and compared their combined z-scores to 0 (50th percentile based on average growth from CNPRC) (S6 Table). BPD and FL were not consistently statistically different from the 50th percentile when combined, while HC (p=0.0004) and AC (p=0.003) were statistically smaller than average overall and throughout several time points. The untreated and mock infected group was also significantly different from average for HC. When comparing the ZIKV-IG/placebo-IG combined group with the untreated/mock combined group, FL was different in a few time points as well as AC and overall AC was significantly different between the two combined groups.

**Antibody dynamics and pharmacokinetics of HIG in pregnant macaques**

ZIKV-IG-treated dams had detectable human ZIKV-specific IgG antibodies by 1 hour post-infusion at 1 dpi that began waning at 2 dpi until a second infusion was performed at 5 dpi as measured by whole-virion binding ELISA (Fig. 3A). Human ZIKV-specific IgG antibodies became undetectable between 13 and 44 dpi. All four ZIKV-IG treated animals had some detectable rhesus ZIKV-specific IgG responses, but only two dams had durable responses detectable after 41 dpi (Fig. 3B). One of the two dams (279087) developed a robust IgG response following viral recrudescence that mimicked the magnitude of response seen in the placebo-IG treated animals by 61 dpi (Fig. 3B). The other ZIKV-IG-treated dam with detectable macaque antibodies after 41 dpi never had detectable virus after 1 dpi. Macaque IgG antibodies were detectable in all placebo-IG treated animals by 13 dpi (Fig. 3C). Placebo-IG treated animals developed similar ZIKV-binding IgG titers as untreated animals yet displayed slower kinetics to peak responses (Fig. 3C).

**Placental pathology was similar between ZIKV-IG and placebo-IG groups**

Placental pathology was scored between groups as described in the main text results. Placental pathology of the HIG-treated groups was compared to a group of animals that were also treated with ferumoxytol and underwent MRI. Placebo-IG treated dams had significantly more placental villous agglutination in disc 1 than dams undergoing MRI (p=0.03) and ZIKV-IG treated dams had more diffuse perivillous fibrin in disc 2 than MRI (p=0.04) and mock animals (p=0.05) (S16 Table). To help parse out whether ferumoxytol played a role in placental pathology, pathology between the two antibody-treated groups was combined and compared to the MRI group. The only feature that was statistically different was diffuse placental perivillous fibrin that was greater in the antibody-treated group in both discs (S16 Table). Scores from subsets of the 21 features were combined to represent fetal and maternal malperfusion (see S12 Table) and were compared between groups. Only fetal placental malperfusion was different between the MRI group and mock-infected dams while no other groups differed from each other or mock infected animals (S17 Table). Maternal malperfusion scores were not different between groups (S18 Table).
